# Differences in spatiotemporal brain network dynamics of Montessori and traditionally schooled students

**DOI:** 10.1038/s41539-024-00254-6

**Published:** 2024-07-10

**Authors:** Paola Zanchi, Emeline Mullier, Eleonora Fornari, Priscille Guerrier de Dumast, Yasser Alemán-Gómez, Jean-Baptiste Ledoux, Roger Beaty, Patric Hagmann, Solange Denervaud

**Affiliations:** 1 Department of Radiology, Lausanne University Hospital and University of Lausanne (CHUV-UNIL), Lausanne, Switzerland; 2 CIBM Center for Biomedical Imaging, Lausanne, Switzerland; 3 Department of Psychology, Pennsylvania State University, University Park, TX USA; 4 MRI Animal imaging and technology, Polytechnical School of Lausanne, Swiss Federal Institute of Technology Lausanne (EPFL), 1015 Lausanne, Switzerland

**Keywords:** Dynamical systems, Education

## Abstract

Across development, experience has a strong impact on the way we think and adapt. School experience affects academic and social-emotional outcomes, yet whether differences in pedagogical experience modulate underlying brain network development is still unknown. In this study, we compared the brain network dynamics of students with different pedagogical backgrounds. Specifically, we characterized the diversity and stability of brain activity at rest by combining both resting-state fMRI and diffusion-weighted structural imaging data of 87 4–18 years old students experiencing either the Montessori pedagogy (i.e., student-led, trial-and-error pedagogy) or the traditional pedagogy (i.e., teacher-led, test-based pedagogy). Our results revealed spatiotemporal brain dynamics differences between students as a function of schooling experience at the whole-brain level. Students from Montessori schools showed overall higher functional integration (higher system diversity) and neural stability (lower spatiotemporal diversity) compared to traditionally schooled students. Higher integration was explained mainly through the cerebellar (CBL) functional network. In contrast, higher temporal stability was observed in the ventral attention, dorsal attention, somatomotor, frontoparietal, and CBL functional networks. This study suggests a form of experience-dependent dynamic functional connectivity plasticity, in learning-related networks.

## Introduction

We face numerous challenges that require innovative mindsets to address them effectively. How can we adopt new perspectives? As Einstein wisely stated, “We cannot solve our problems with the same thinking we used when we created them.” Shaping new mindsets is essential, and education offers a promising means to achieve this. Early life experiences significantly influence our thinking and adaptability later in life, largely due to experience-dependent neural plasticity^1^. Consequently, pedagogy holds the potential to facilitate lasting societal changes. While many studies have examined the impact of pedagogy on behavioral outcomes, there has been comparatively less focus on its effects on underlying brain dynamics.

In contemporary school settings, various pedagogies are implemented. The most common is known as ‘traditional’ pedagogy, characterized by classrooms with same-aged students, teacher-provided instruction, and evaluations that focus on memory and recall^2^. Students primarily learn from teacher-based feedback. While Western societies have mainly adopted a traditional pedagogy for their public schools, alternative pedagogies, such as Montessori, exist. Montessori schools, numbering >15,700 worldwide^3^, feature mixed-age classrooms and peer-to-peer learning. The curriculum emphasizes student-led, trial-and-error learning without external feedback, grades, or tests. Montessori classrooms utilize self-corrective materials, allowing children to choose their learning activities. Also, there is one teacher for a large group of students to encourage social interaction^4^.

Comparative studies between Montessori and traditional pedagogy have identified differences in academic, cognitive, and social-emotional outcomes^5–8^. Montessori students tend to perform better in academic, creative, and socio-emotional tasks^7,9,10^. They also demonstrate improved self-regulation skills and constructive social interactions^9,11,12^. Quantitative behavioral studies have been complemented by recent work examining differences in brain activity and functional connectivity. For example, during a functional magnetic resonance imaging (fMRI) math task, Montessori students exhibited increased neural activity in the right parietal and frontal regions and greater functional connectivity between the anterior cingulate cortex (ACC) and frontal regions after incorrect trials. Conversely, traditionally schooled students displayed increased functional connectivity between the ACC and hippocampus following correct trials^13^, suggesting distinct learning strategies.

In another study on creative thinking—comparing Montessori and traditionally schooled participants’ dynamic and static functional connectivity—Montessori students consistently scored higher on creativity tasks and spent less time in an intra-connectivity default mode network (DMN) state. The results also suggested a modulatory effect of pedagogy on the salience network (SN), which is central to cognitive flexibility and creative thinking processes. Lower creativity-related brain network fluidity in traditional students may be due to a strengthened SN over other networks like the executive control network (ECN) and DMN^14^. Such neural differences imply that spatiotemporal brain dynamics within all functional systems might be impacted by the learning experience. However, the extent to which school experiences potentially influence the development of brain network dynamics remains to be fully understood.

**Fig. 1:**
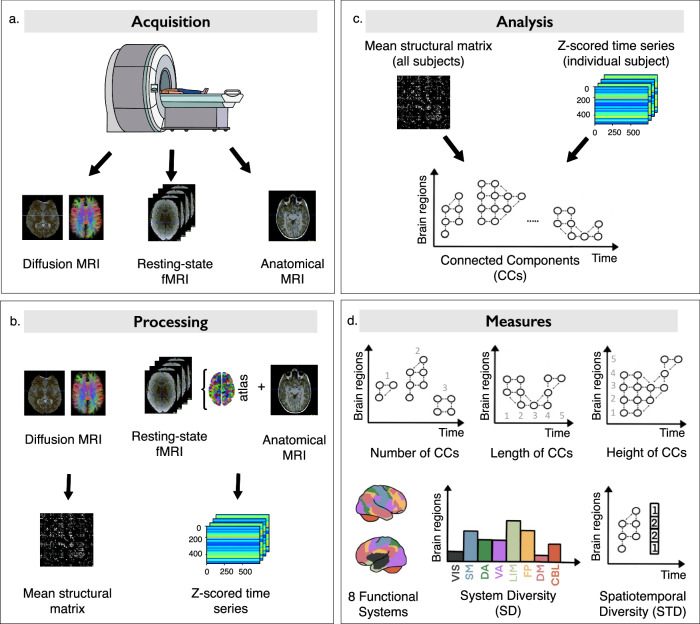
The spatiotemporal connectome framework. **a** Data acquisition. Diffusion MRI, resting-state fMRI, anatomical MRI. **b** Processing. Creation of the mean structural connectivity matrices and functional time series. **c** Spatiotemporal connectome. By merging the mean structural connectivity matrix, created with all the subjects, with the functional activations (point process analysis) of each subject, a multilayer graph was created: the spatiotemporal connectome. **d** System diversity (SD) and spatiotemporal diversity (STD). Each brain region is associated with a functional system: visual (VIS), somatomotor (SM), dorsal attention (DA), ventral attention (VA), limbic (LIM), frontoparietal (FP), default mode (DM), and cerebellar (CBL) systems.

Recent advances in network neuroscience methods have offered new perspectives on the mean functional connectivity and the temporal neural dynamic. One recent method for the investigation of the latter is the spatiotemporal connectome^15^. This analytical framework characterizes brain functional connectivity with a structural prior, enabling the extraction of the most robust and direct connections. Neural activity over the resting-state acquisition is chunked into successive time windows where mean functional connectivity constrained to structural connectivity per brain regions (i.e., atlas-based) is computed (Fig. 1b). These connectivity matrices (506 × 506 brain regions) are then translated into Connected Components (CCs), plotted as a function of time (*x* axis) and space (*y* axis) (Fig. 1c). CCs can be quantified in various ways: in terms of number (i.e., the amount of neural activity), length (i.e., the average duration of neural activity, indicating whether it is prolonged in time), and height (i.e., the ratio of brain regions involved in a process, regardless of functional systems) (Fig. 1d). However, this characterization remains somewhat limited in its interpretation as it does not allow us to relate these metrics to known brain functions. To alleviate this limitation, Vohryzek et al.^16^, translated CCs into more complex measures by integrating a classifier of the 7 functional systems of Yeo’s atlas^17^. These group-level metrics of network properties are system diversity (SD) and spatiotemporal diversity (STD). Thanks to these two metrics, we can characterize spatiotemporal brain dynamics and compare values between groups. First, in terms of integration and segregation (SD) that informs us about the ability to complexify cognitive processes. Second, in terms of temporal stability (STD), that informs us about the ability to maintain cognitive engagement over time. These two measures have been studied across development in these forms, or slightly different ones, reporting that both integration-segregation processes and stability in neural activity increase with age^16^. For example, a recent comparative study between children and adults reported functional reorganization of brain spatiotemporal dynamics, with maturing brains becoming more functionally integrative (increased global SD) and gaining stability within neural systems (decreased global STD)^16^. These changes parallel experience-dependent plasticity, where the brain is shaped by experience^18–20^. This process has been studied in the context of sensory processing, language acquisition, and social bonding^21^.

Using these measures, the current study further investigated whether schooling experiences through different pedagogies would modulate brain network dynamics differently, adopting a cross-sectional perspective. We examined the spatiotemporal brain network dynamics of 87 students from Montessori and high-quality traditional schools, using resting-state fMRI and diffusion-weighted imaging to capture both functional and structural brain connectivity.

The Montessori pedagogy promotes trial-and-error learning, allowing students to seek knowledge independently and engage multiple senses with self-corrective materials^4,22^. In contrast, the traditional school system emphasizes paper-based work, grades, teacher instructions, and value-based feedback (e.g., grades, reward, punishment). Research has shown that Montessori students tend to possess a higher capacity for creative thinking, knowledge integration, and self-monitoring^6–8,13,23,24^. These findings suggest higher self-direction that could be reflected in differences in spatiotemporal brain dynamics. Given that across development, changes in connectome topology (i.e., brain network dynamics’ maturation) tend to converge toward decreased segregation and increased integration^25^, we hypothesized that Montessori students would exhibit higher global SD (integration) and lower global STD (increased neural stability) compared to traditional students^16^. These patterns would be visible in functional systems involved in the regulation and coordination of behavior, working memory, problem-solving, and cognitive operations, such as the frontoparietal (FP), somatomotor (SM), dorsal attention (DA), ventral attention (VA)^17^, and cerebellar (CBL)^26,27^ functional systems.

## Results

### Group variables

Potential group differences in terms of demographics, fluid intelligence ability, and familial environments were first evaluated. Importantly, no significant differences were observed concerning age, familial socioeconomic status (SES), parental pedagogy interest, or fluid intelligence (all *ps* > 0.06). Furthermore, both groups exhibited comparable age distribution (F(1,85) = 0.147, *p* = 0.702) and comparable number of years of schooling within a pedagogical context (*U* = 845, *p* = 0.393). Number of years enrolled within a schooling system correlated positively with participant’s age (*r*(85) = 0.859, *p* < 0.001) (Supplementary Fig. 2). Additionally, the Chi-squared test revealed no significant difference in gender ratio between the two groups (*p* = 0.35) (see Table 1). We report no differences between Montessori- and traditionally schooled participants on these variables for this dataset, indicating between-group homogeneity in terms of age, gender, family environments, and intelligence level.

**Table 1:** Demographics and group variables

Control variable	Montessori (*n* = 42)	Traditional (*n* = 45)	Overall (*n* = 87)	Statistics
Age mean (st dev)	9.92 (2.97)	10.10 (2.83)	10.02 (2.88)	U(87) = 906, *p* = 0.75, *d* = −0.04
Age min, max	4.60, 18.00	5.20, 17.20	4.60, 18.00	
Gender ratio (f/m)	22/20	28/17	50/37	Chi2 = 0.86, *p* = 0.35
Socioeconomic status	3.04 (0.51)^(1)^	3.07 (0.65)^(2)^	3.05 (0.58)^(3)^	U(87) = 850, *p* = 0.78, d = −0.04
Pedagogy interest	2.76 (0.44)^(4)^	2.51 (0.70)^(5)^	2.64 (0.59)^(6)^	U(87) = 744, *p* = 0.13, d = 0.16
Fluid intelligence	32.67 (4.14)	32.77 (3.26)^(7)^	32.53 (3.99)^(8)^	U(87) = 837, *p* = 0.45, d = −0.09

Statistical comparison of age, gender, socioeconomic status, pedagogy interest, and fluid intelligence data between the Montessori-schooled participants and traditionally schooled participants. Missing data: (1) one, (2) two (3) three, (4) one, (5) two, (6) three, (7) one, (8) one.

### Spatiotemporal connectome measures

**Fig. 2:**
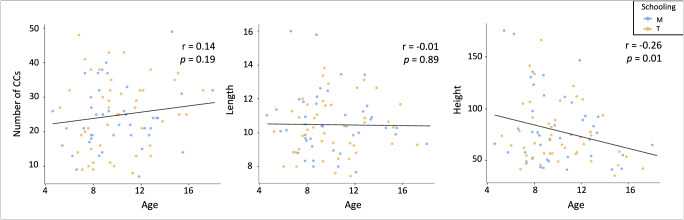
Number of CCs, length, and height. Comparison of the number of CCs, length, and height values of Montessori-schooled students (M) and traditionally schooled students (T), according to age. Neither schooling nor age exhibit a significant effect on participants’ number of CCs nor their length. A significant effect of age on the height of the CCs was found (F(1, 83) = 5.99, *p* < 0.016).

Our primary objective was to examine whether different pedagogical experiences influence brain network dynamics, which are crucial for students’ cognitive and emotional development. First, we investigated the interplay between individual key characteristics of the CCs (number, length, height), and related demographic variables (age and pedagogy), to discern potential differentiations within the two groups of students as a function of development. Neither the number of CCs, nor their length was related to participants’ age, schooling experience (i.e., pedagogy), nor their interaction term (all *ps* > 0.211) (Supplementary Table 1). These outcomes suggest that neither schooling nor age exhibits a significant effect on participants’ number of CCs or their length. However, the analysis revealed a significant effect of age on the height of the CCs (F(1, 83) = 5.99, *p* < 0.016). These results underscore that age plays an important role in the spatial extent (height) of the CCs variation, regardless of schooling experience. Both Montessori-schooled and traditionally schooled students exhibited similar characteristics, indicating a decrease across development (Fig. 2). This suggests a significant reorganization of the spatiotemporal neural activity across development.

**Fig. 3:**
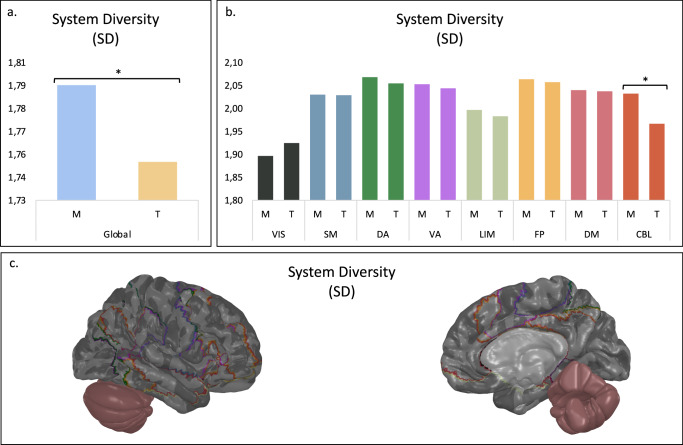
SD at the global and 8 functional systems levels. Comparison of SD values between Montessori-schooled students (M) and traditionally schooled students (T). Each brain region is associated with a functional system: visual (VIS), somatomotor (SM), dorsal attention (DA), ventral attention (VA), limbic (LIM), frontoparietal (FP), default mode (DM) and cerebellar (CBL) systems. **a** SD value for the global level (*p* = 0.017). **b** SD values for the eight individual functional systems; only the CBL differed (*p*_FDR_ < 0.001). **c** Functional systems with significant differences between Montessori-schooled students and traditionally schooled students are colored for SD.

**Fig. 4:**
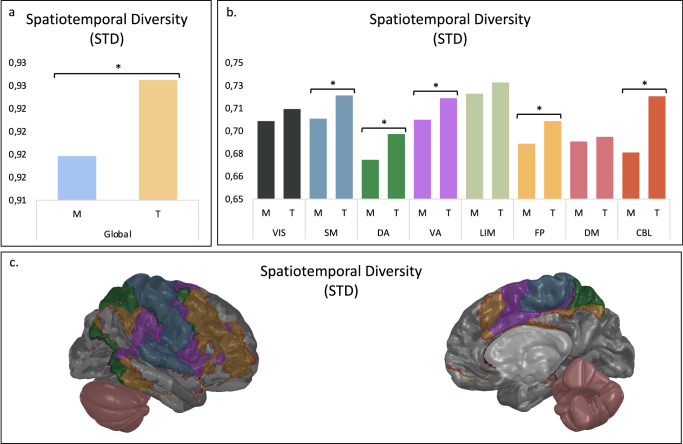
STD at the global and 8 functional systems level. Comparison of STD values between Montessori-schooled students (M) and traditionally schooled students (T). Each brain region is associated with a functional system: visual (VIS), somatomotor (SM), dorsal attention (DA), ventral attention (VA), limbic (LIM), frontoparietal (FP), default mode (DM) and cerebellar (CBL) systems. **a** STD value at the global level (*p* = 0.013). **b** STD values for the eight individual functional systems; the DA (*p*_FDR_ = 0.020), VA (*p*_FDR_ < 0.001), SM (*p*_FDR_ < 0.001), FP (*p*_FDR_ = 0.020), and CBL (*p*_FDR_ = 0.020) functional systems differed. **c** Functional systems with significant differences between Montessori-schooled students and traditionally schooled students are colored for STD.

To further investigate this reorganization specifically as a function of schooling experience (Montessori, traditional) in terms of space and time, we assessed the SD (integration) and STD (stability) values of resting-state brain networks contrasting Montessori and traditionally schooled students. By comparing empirical to null distributions, we found significant differences between groups at the whole-brain level for both SD and STD. Montessori-schooled students exhibited higher SD (greater cross-system integration) (*p* = 0.017) (Fig. 3a) and lower STD (higher neural stability) (*p* = 0.013) (Fig. 4a) at the global level.

We further investigated the eight functional systems and discovered significant results for several large-scale brain networks. For SD, significant differences were observed in the CBL functional system, with Montessori-schooled students having higher values than traditionally schooled students (*p*_FDR_ < 0.001) (Fig. 3b, c). No group differences were found in any other functional networks (*p*_FDR_ > 0.496). Regarding STD, significant differences emerged between Montessori- and traditionally schooled participants in the DA (*p*_FDR_ = 0.020), VA (*p*_FDR_ < 0.001), somatomotor (*p*_FDR_ < 0.001), FP (*p*_FDR_ = 0.020), and CBL (*p*_FDR_ = 0.020) functional systems (Fig. 4b, c). Montessori-schooled participants consistently exhibited lower STD than traditionally schooled participants. However, no between-group differences were observed for the visual, limbic, and default mode functional systems (*p*_FDR_ > 0.110).

## Discussion

Increasing evidence shows that school pedagogy modulates cognitive and social-emotional skills, but its influence on neural networks remains unclear. Considering the importance of spatiotemporal brain dynamics for learning processes, this study examined the differences in brain network dynamics between Montessori- and traditionally schooled participants.

Groups were comparable in terms of age (i.e., mean age and age distribution), gender ratio, fluid intelligence scores, families’ SES, and parental interest in pedagogy. Furthermore, the individual’s overall count of distinct clusters or regions within the brain that exhibit synchronized or correlated neural activity (i.e., CCs) was characterized in terms of number, length, and height. Participants’ number of CCs and their length did not differ as a function of age, pedagogy, or schooling experience across development (i.e., age X pedagogy). However, individual CCs’ height was related to age; across development, a shortening of the CCs was observed. This suggests a robust functional reorganization of neural activity that is overall not influenced by schooling experience. However, to investigate subtle reorganization in terms of spatial and temporal metrics as a function of schooling experience, we contrasted Montessori-schooled participants with traditionally schooled participants on spatiotemporal metrics. Montessori-schooled participants exhibited overall higher cross-functional system integration (greater SD) and increased neural stability (lower STD) compared to their peers from traditional schools. This suggests that the educational approach may influence specific brain dynamics (SD and STD), but not the specific structural attributes of the CCs.

Building on previous work, the findings for SD and STD suggest a more adult-like pattern of brain spatiotemporal dynamics in Montessori-schooled students compared to traditionally schooled students^16^. Interestingly, functional networks related to these effects included specific functional networks. Higher neural stability (STD) was observed in Montessori- compared to traditionally schooled participants in their VA, DA, SM, FP, and CBL functional networks.

The results observed for DA and VA networks are particularly interesting in the context of education, as they are reliably involved in flexible attentional control^28^. The VA network typically responds to unexpected but behaviorally relevant stimuli, while the DA network is implicated in attentional subprocesses such as top-down controlled attentional selection^29,30^. These networks undergo plastic changes across development^31^ and seem permeable to exercises and experience^32^. Our observations could relate to the different means used to guide attention in Montessori which involves repetitive object manipulation, self-direction, and uninterrupted working hours, versus traditional pedagogy. Our findings corroborate outcomes from a recent meta-analysis, reporting that the Montessori pedagogy supports better children’s attention^22,33^. We hope this preliminary evidence will stimulate the interest of other research groups to investigate further VA and DA dynamics as a function of schooling experience.

We further report higher stability of the SM functional system in Montessori-schooled participants compared to traditionally schooled participants. As the brain’s transducer, the sensorimotor network is responsible for sensing and processing external stimuli to generate an appropriate behavioral response^34^. Corroborating this observation, a recent review about Montessori versus traditional education reports comparative studies where learning by doing, moving, and using multiple senses^4,35^ is more efficient for learning and retaining information than traditional approaches mostly based on auditory and visual stimuli^36^.

We further report higher neural stability in the FP functional system for Montessori-schooled participants compared to traditionally schooled participants. The FP network is involved in coordinating whole-brain network activity and is crucial for flexible interaction with other control and processing networks^37^. Additionally, the FP network is involved in executive skills and processes such as working memory, attention focusing, and regulating goal-directed behavior^38^. One possibility to explain these findings is that a self-directed curriculum like Montessori, which emphasizes trial-and-error learning strategies, engages higher neural dynamics related to executive abilities (FP). The pattern observed in Montessori-schooled participants is consistent with previous studies reporting a positive effect on academic outcomes and executive measures^5–7,9^. Moreover, recent findings showed differences in error and performance monitoring processes between Montessori and traditional-school students, also reflected in higher prefrontal activity^8,13^.

Finally, higher cross-system integration (SD) and neural stability (STD) were observed within the CBL functional system for Montessori-schooled participants compared to traditionally schooled participants. Recent work suggests that motor and cognitive development are closely related; studies have shown that CBL function extends beyond motor control^39^ and is associated with cognition^27^, involving basic cognitive operations such as language, implicit and explicit learning, and memory processes^26,40^. CBL functions are like those of the dorsolateral prefrontal cortex^41^. The link between fine motor development and academic performance has been demonstrated in studies showing that fine motor control in kindergarten predicts subsequent academic performance in math and literacy^42–44^. Additionally, developmental delays in fine motor development are linked to later educational and social difficulties at school^45^. As the CBL functional system is connected to both motor control and cognition, the higher maturation of this network in Montessori-schooled participants could be related to active-based self-directed pedagogical practices. However, more work is needed to better understand this robust effect found in the CBL functional system.

Notably, local differences were localized for the SD metric (integration), while the STD ones (stability) were more spread. Various developmental studies focusing on functional and structural connectivity maturation have found that structural networks mature from spatially proximal to more integrative topologies, supporting the functional reorganization of increasingly complex higher-order cognitive interactions between various resting-state networks^16,46–48^. Specifically, during childhood and adolescence, changes in connectome topology converge toward decreasing segregation and increasing integration^25^. One possible interpretation of our results highlighting more differences and changes in local STD metrics is that network stability (as captured by STD) develops slightly before between-functional system coordination (as captured by SD). Further research is needed to investigate the protracted nature of brain development and, more precisely, the temporal development of integration and stability of functional systems.

Our study has some limitations. First, despite our efforts to equate groups on demographic and cognitive factors, the non-randomized design tempers broader generalization of the findings beyond our sample. Additionally, there may be differences between Montessori and traditional-school families in various aspects, beyond SES, such as cultural influences, or openness to new experiences, which could have influenced the observed outcomes. Therefore, it is essential to acknowledge these limitations when interpreting the results and consider them for future work. Indeed, other factors than pedagogy (e.g., family, motivation) may also contribute to the differences we see between the groups (e.g., cognitive flexibility of parents). However, we are confident that the major factors have been considered, and the effects we have observed are partly indicative of the school experience.

The participants were selected from a specific geographical area, based on the city’s official statistical data on mean salary, reflecting an upper-class-salary population. It would be interesting to investigate other regions and different populations, such as middle or lower class, to see if the same results are obtained. In addition, exploring the characteristics of teachers (e.g., number of years of training, and number of years of experience) could provide valuable insights. Future work should replicate this study, while we will complement ours with longitudinal data points in a few years to overcome the current limitation of cross-sectional data preventing causal claims. Also, future work should examine specific pedagogical features of Montessori education and their impacts on spatiotemporal brain dynamics, as there must be specific variables in Montessori pedagogy that can explain these effects. We suggest testing multisensory processes and movement-based learning, based on our observations of the sensorimotor and cerebellar functional systems. Finally, the measures we obtained for SD and STD are group-level measures and are not defined at the subject level for the number, length, and height of the CCs. Analyzing CCs from a single participant may not capture the diversity of all functional systems, preventing the measurement of SD for each functional system due to a lack of statistical power. Consequently, SD and STD measures require enough CCs for a reliable outcome, leading to their calculation on a group-level rather than individually. This limitation prevents us from correlating brain metrics with behavioral measures in this study.

Despite these limitations, our study provides preliminary evidence of differences in brain dynamic connectivity as a function of pedagogy experienced. Our findings contribute to understanding how early and protracted experiences like education shape the development of human brain network dynamics. Considering the importance of flexible brain network dynamics in adulthood, we hope these findings will motivate future cross-disciplinary work bridging network neuroscience, psychology, and pedagogy. Implementing and combining these approaches may reveal new aspects of neurodevelopment with direct implications for educational practices.

## Methods

### Participants

Between 2018 and 2021, students from Montessori or traditional schools were recruited as part of a large study investigating the effects of pedagogy on child development (Supplementary Fig. 1). Inclusion criteria for this study were age (from 4 up to 18 years old) and pedagogical background (a child had to experience the same pedagogy—Montessori, traditional—from the age of 4, or for at least the past 3 years). Exclusion criteria were learning or behavioral disabilities, as reported by their parents in a tailor-made questionnaire. Recruitment was made in more than 20 different schools. Montessori private schools were selected based on criteria set by the International Montessori Association (https://montessori-ami.org). Since Montessori schools in Switzerland are only private systems, a specific geographical area was chosen according to the city’s official statistical data on mean salary to match with upper-class-salary traditional schools. Additionally, to evaluate homogeneity between participants from the two pedagogies, data on fluid intelligence, parental SES, and parental interest in pedagogy were collected. Participants with learning disabilities and sensory impairments, as reported by the parents, were excluded from the analyses. The study protocol was approved by the local ethical committee (Commission d’Ethique Romande Vaud, CER-VD 2018-00244) and conducted following the Declaration of Helsinki. Written informed consent to participate and to publish was obtained from parents, and oral informed consent was obtained from each child.

A total of 100 healthy participants underwent magnetic resonance imaging (MRI). Data from 13 participants were excluded from the study due to motion artifacts during the MRI session or dental braces interferences, resulting in data from 87 participants for the analyses (50 females; aged 4.60–18.00 years, mean ± standard deviation (stdev) = 10.02 years old ± 2.88; 42 Montessori-schooled participants).

### Group variables

Given a possible selection bias due to Montessori-schooled participants being enrolled in private and not public schooling systems, different variables were collected to test between-group homogeneity on the following factors: (i) age; (ii) gender; (iii) SES; (iv) pedagogy interest of the parents; and (v) fluid intelligence. These data were collected on the same day before or after the scanning session.

- SES was assessed using a paper-based parental questionnaire that inquired about professional situation, category, and education level. Higher scores denoted higher SES, with 4 being the maximal score^49^.
- The pedagogy interest score was assessed using a questionnaire about parental interest in education and pedagogy (e.g., the number of books about education at home) and their parenting style. Higher scores denote greater involvement in the child’s intellectual development and increased pedagogy knowledge. The questionnaire was completed by 95.5% of the parents of traditionally schooled students and 97.6% of the parents of Montessori-schooled students.
- Fluid intelligence level was evaluated using the short black-and-white version of the Raven’s Progressive Matrices test (PM-47). The Raven’s Progressive Matrices provide a nonverbal assessment of intelligence and measure two complementary components of general intelligence: reproductive ability (the capacity to store and reproduce information) and deductive ability (the capacity to think clearly and make sense of complex data) (Raven & Court, 1998). The PM-47 test consists of 36 items in the form of an incomplete matrix with several possible answers. Participants had 15 min to answer. One point was given for each correct response, with the final score being the sum of all correct answers (maximum 36). A higher score denotes a higher level of fluid intelligence.

### MRI data acquisition

A Siemens (Healthineers, Erlangen, Germany, VE11E Software version) 3 T Prisma-Fit MR scanner with a 64-channel head coil in the Lemanic2 CIBM Center for Biomedical Imaging of the Lausanne University Hospital (CHUV) was used to obtain the MRI data. Each subject underwent an MRI session that consisted of three different acquisition protocols (Fig. 1a): (i) 3-dimensional T1-weighted (T1w) MP-RAGE (Magnetization Prepared Rapid Acquisition Gradient-Echo), (ii) a diffusion MRI (dMRI) acquired with diffusion spectrum imaging (DSI) sequence, and (iii) a 6-minute resting-state fMRI.

The T1w images were acquired in a sagittal orientation with echo time (TE) = 2.47 ms, repetition time (TR) = 2000 ms, inversion time (TI) = 900 ms, flip angle (FA) = 8°, field of view (FOV) = 256 × 256 mm^2^ and voxel size = 1 × 1 × 1 mm^3^.

Diffusion-weighted images were acquired using a fat saturation Echo Planar Imaging (EPI) sequence with simultaneous multi-slices (SMS) factor 6, TE = 80 ms, TR = 5200 ms, flip angle = 90°, bandwidth (BW) = 1630 Hz/Px, acceleration factor PE = 3, acceleration factor slice = 2, FOV = 239 × 239 mm^2^, voxel size = 1.6 × 1.6 × 1.6 mm^3^ and *b* values = 0, 700, 1000, 2000 and 3000 s/mm^2^. Ten non-diffusion-weighted (*b* = 0 s/mm^2^) and 137 diffusion-weighted images were acquired along gradient directions distributed over a unit radius sphere (16, 26, 47 and 48 images for *b* = 700, 1000, 2000 and 3000 s/mm^2^, respectively).

The fMRI data were acquired from 720 continuous measurements using a fat saturation standard EPI echo sequence, covering the whole brain with 48 contiguous axial slices, an isotropic voxel size of 2, 4 mm^2^, TR = 500 ms, echo time TE = 33 ms, flip angle = 80°, matrix size = 94 × 94, FOV = 224 × 224 mm^2^, acceleration factor slice = 8, BW = 2418 Hz/Px, and EPI factor = 94.

To prevent head motion, foam pads were placed around the head of the subject inside the coil. For the resting-state fMRI acquisition, participants were asked to fix a cross on the screen, let their minds wander, and not fall asleep.

### Structural connectivity template

Anatomical and diffusion MRI images were processed using the Connectome Mapper 3 pipeline^50^ to create the structural connectivity template.

First, the individual T1w images generated accurate cortical surfaces (white and pial) and individual cortical parcellations^51,52^. A symmetric version of the method developed by Cammoun et al.^53^ was used to perform a multi-scale symmetric parcellation of the cortical surface, starting with the 34 regions (per hemisphere) described in Desikan et al.^52^.

This multi-scale cortical parcellation was combined with hippocampus subfields, brainstem parcellations^54,55^, and atlas-based segmentation of thalamic nuclei^56^ to obtain five individual gray-matter (GM) parcellations for each subject. The resulting multi-scale parcellation contains 128, 174, 276, 506, and 1062 GM regions for scales 1, 2, 3, 4, and 5, respectively, but only the fourth scale was selected for all the analyses, based on previous work^16^.

Secondly, individual DSIs were preprocessed and employed to estimate the white matter (WM) streamline distribution. The constrained spherical deconvolution^57^ algorithm was used to compute the intravoxel fiber distribution functions employed by the deterministic tractography approach (SDstream)^58^, to calculate the WM streamlines. The final structural connectivity matrix (Fig. 1b) represents the overall set of streamlines connecting cortical and sub-cortical region pairs (matrix of dimensions (506 × 506)). Individual matrices were merged into one by averaging them to obtain a mean structural connectome matrix.

### MRI Processing

Imaging data were preprocessed using fMRIPrep 20.2.1^59^, based on Nipype 1.5.1^60^. First, anatomical data were preprocessed using the Freesurfer pipeline^51^, where T1w images were corrected for intensity non-uniformity and then used as a T1w reference throughout the workflow. Second, preprocessing was applied to the functional data. A reference volume and its skull-stripped version were generated. Transforms to correct head motion were applied to the blood oxygenation level-dependent (BOLD) time series to resample it onto their original native space. Non-steady-state volumes and spatial smoothing were removed, and then, motion artifacts on the preprocessed BOLD were removed using independent component analysis on the MNI space-time series. More precisely, the AROMA correction method was used to remove motion-related artifacts from fMRI data, as it allowed the reduction of motion-induced signal variations in fMRI data^61^.

Based on the preprocessed fMRI images, several confounding time series were extracted: framewise displacement (FD), the spatial standard deviation of successive difference images (DVARS), and three region-wise global signals. Visual inspections of the preprocessed and post-processed data were done to select the data. Included participants did not present excessive movements (mean DVARS and FD < 1.5), FD (mean = 0.27, SD = 0.26), and DVARS (mean = 1.11, SD = 0.07) (for more detailed information, see SI).

An average fMRI signal was computed for an atlas of 506 parcellations^15^ using a homemade Python script (Python Software Foundation. Python Language Reference, version 3.5) (Fig. 1b).

### Spatiotemporal connectome

A multilayer graph was created to represent the brain’s functional dynamics (the preprocessed fMRI signal) constrained by the structural connectivity, generating a spatiotemporal connectome^15^. This unified framework shows the functional relations between anatomically connected brain regions, with each layer corresponding to a single time point of functional activity. To capture the temporal dynamics of functional brain activity, point process analysis^62^ was applied to the fMRI time series of the 506 regions of the parcellation. The time series were z-scored and then thresholded to be reduced to binary point processes, indicating the active status of brain region *i* at time point *T*. The threshold was chosen by Griffa et al.^15^ as 2 stdev. By merging the point processes (active status of the regions) and the anatomical connectivity information, a multilayer graph was created. Each node of this graph represents a specific brain region at a given time point. Connections were created if two brain regions (nodes) were both functionally coactive and structurally connected (linked by a WM tract) within a specific or two consecutive time frames. This graph encodes weakly CCs (i.e., the count of distinct clusters or regions within the brain that exhibit synchronized or correlated neural activity), representing transient networks of spatiotemporal connectivity (Fig. 4c). They extend spatially (across different brain regions) and temporally (over multiple time points). This multilayer graph allows following the propagation of brain dynamics on the structural connectome, as it links functional co-activation of anatomically connected brain region^15^.

### Spatiotemporal connectome measures

The CCs can be described as a spatial activation map by defining a vector *x* where *x* = [x1,x2,…,xN], N is the number of brain regions, and xi is the number of time points within a CC where brain region i is active. For each entry of the spatial activation vectors of the CCs, a functional system label f was associated (according to the classification of Yeo et al.^17^, and the CBL functional system). These CCs can be used as markers of global brain connectivity and represent an expression of brain communication processes, as they reveal time-varying pathways of activity propagation and, more precisely, characteristic spatiotemporal patterns of functional activity propagation. Through their clustering, they show the spreading of functional events, across anatomically connected brain regions, and at specific time points, and allow to see a variety of functional configurations, along with static anatomical substrate^15^. By examining the number of CCs, we can reveal information about the organization of neural networks in the brain. Using the length and the height metrics allowed us to gain insights into the temporal duration of the component’s existence and their spatial extent, respectively. Then, using the spatial activation vectors of the CCs, two other measures of brain dynamics were defined: SD and STD (Fig. 4d). First, SD quantifies the number of functional systems activated simultaneously for a given time window. It is defined as a functional system histogram-based entropy measure and represents the distribution of the CBL and the seven Yeo’s functional systems for each CC: VIS, SM, DA, VA, LIM, FP, DM, and CBL systems. Second, STD quantifies the temporal stability of functional system activation across time. It represents the average cosine similarity between vector-embedded CCs and allows the quantification of the dynamic spatiotemporal polyvalence of the different brain regions.

Using the individual multilayer graphs, the CCs of the subjects were extracted, and SD and STD were calculated following the method of Vohryzek et al.^16^. The CCs were concatenated for group comparison, and the SD and STD were computed at two different spatial scales: a global scale and a functional system scale. For the global investigation scale, all the CCs extracted from a set of spatiotemporal connectomes were considered. For the functional system scale, all the CCs with at least 20% of brain regions labeled according to a certain functional system f were attributed to this specific functional system and SD and STD were estimated for each system and each group.

Individual measures of CCs (number, length, and height) were employed, but analyzing CCs from a single participant may not adequately represent the diversity of functional systems. Therefore, SD and STD measures would require a sufficient number of CCs for a reliable outcome, leading to their calculation on a group-level rather than an individual level.

The analyses with these measures allow us to compare and contrast the brain dynamics of individuals from Montessori and traditional schools, examining how their respective educational environments might influence the organization, activation, and temporal stability of the different functional systems in the brain.

## Statistical analyses

### Group variables

As Montessori schools in Switzerland are exclusively private institutions and traditional schools are public, several cognitive and family-related variables were collected to ensure homogeneity between Montessori and traditionally schooled participants. To test the normality of the data, a Shapiro–Wilk test was conducted. Based on the results, Mann–Whitney *U* tests were applied to age, SES, pedagogy interest, fluid intelligence, and years in schooling within pedagogical system variables to examine any statistical differences between the two groups. For the gender variable, a Chi-squared test was used to compare Montessori and traditionally schooled participants. All statistical analyses were performed using the Jamovi tool.

### Spatiotemporal connectome measures

First, three analyses of covariance were computed to elucidate the interplay between each CC’s characteristic (i.e., number, length, and height), and demographic factors of pedagogy and age. The CCs metrics served as the dependent variable, pedagogy (Montessori, traditional) as the fixed factor, and age as the covariate, we further included the interaction term between age and pedagogy.

Second, for both Montessori and traditionally schooled groups, SD and STD values were computed at the global scale (whole-brain level) and the functional systems level. *P* values were assessed based on the randomized distribution of SD and STD values, utilizing 1000 permutations of subjects between the two groups and a two-tailed permutation test (bootstrapping). At the functional systems level, SD and STD values were calculated for each of the eight functional systems. The functional system-based analysis results were corrected for multiple comparisons using FDR correction, and p-values were considered significant if *p* < 0.05.

### Reporting summary

Further information on research design is available in the Nature Research Reporting Summary linked to this article.

### Supplementary information

Supplementary Information

Reporting Summary

## Data Availability

Neuroimaging derivatives (CC values, height, length, STD, and SD measures) are shared as supplementary material.

## Code availability

Open-source codes were used for the analyses, see Vohryzek et al.^16^.
