# Supplementary material for: Differences in spatiotemporal brain network dynamics of Montessori and traditionally schooled students
Source: NPJ Sci Learn. 2024 Jul 10;9:45. doi: 10.1038/s41539-024-00254-6 (PMC11236971; doi:10.1038/s41539-024-00254-6)
Supplement: Supplementary file 1 — Supplementary Information [file 41539_2024_254_MOESM1_ESM.pdf]

Supplementary Figures and Tables

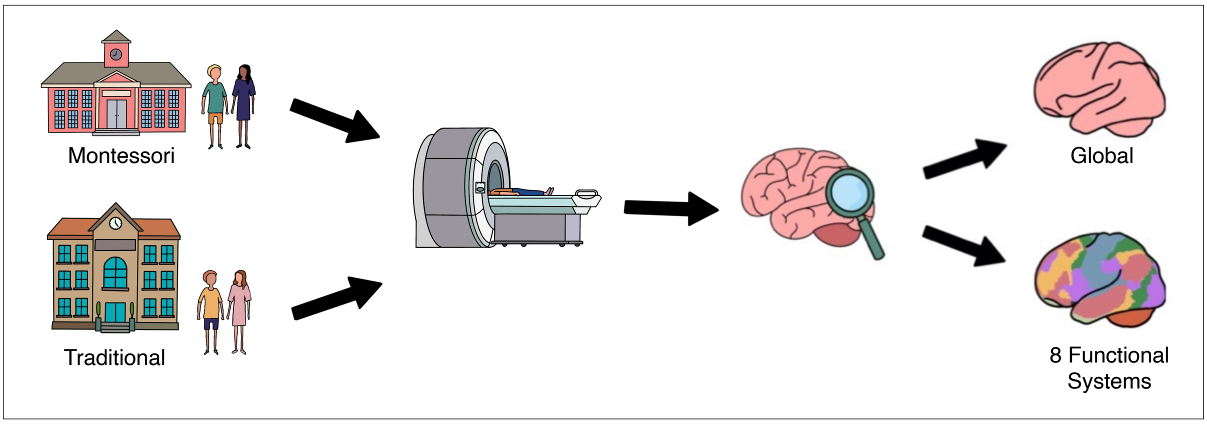

Supplementary Figure 1. Study protocol. Students from high-quality traditional and Montessori schools were recruited for this brain imaging study. Each child from Montessori or traditional pedagogy underwent MRI examination, allowing the quantification of temporal and spatial network dynamics as a function of schooling experience through a cross-sectional design.

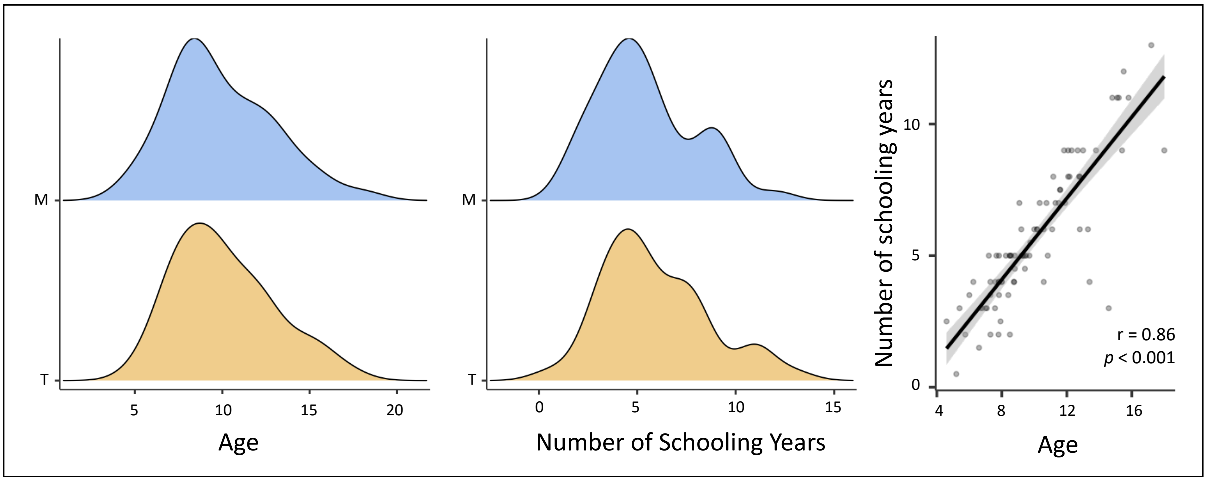

Supplementary Figure 2. Age distribution and number of schooling years of Montessori-schooled students (M) and traditionally-schooled students. Both groups exhibited comparable age distribution ($F(1,85) = 0.147$, $p = 0.702$) and comparable number of years of schooling within a pedagogical context ($U = 845$, $p = 0.393$). The number of years enrolled within a schooling system correlated positively with participant's age ($r(85) = 0.859$, $p < 0.001$).

ANCOVA		Number of CCs			Height			Width		
	DF	F	*p*	η²p	F	*p*	η²p	F	*p*	η²p
Age	1	1.5912	0.211	0.019	5.999	0.016	0.067	0.00754	0.931	0.000
Schooling	1	0.0244	0.876	0.000	0.420	0.519	0.005	1.45734	0.231	0.017
Schooling ✻ Age	1	8.82e-8	1.000	0.000	0.165	0.686	0.002	1.00809	0.318	0.012

Supplementary Table 1. Results of the ANCOVA for the number of CCs, Height and width related to participants' age, schooling experience.

Supplementary Methods

Anatomical data preprocessing

A total of 1 T1-weighted (T1w) images were found within the input BIDS dataset. The T1w image was corrected for intensity non-uniformity (INU) with N4BiasFieldCorrection [1], distributed with ANTs 2.3.3 [2]RRID:SCR_004757), and used as T1w-reference throughout the workflow. The T1w-reference was then skull-stripped with a Nipype implementation of the antsBrainExtraction.sh workflow (from ANTs), using OASIS30ANTs as target template. Brain tissue segmentation of cerebrospinal fluid (CSF), white-matter (WM) and gray-matter (GM) was performed on the brain-extracted T1w using fast (FSL 5.0.9, RRID:SCR_002823, [3]. Brain surfaces were reconstructed using recon-all (FreeSurfer 6.0.1, RRID:SCR_001847, [4], and the brain mask estimated previously was refined with a custom variation of the method to reconcile ANTs-derived and FreeSurfer-derived segmentations of the cortical gray-matter of Mindboggle (RRID:SCR_002438, [5]. Volume-based spatial normalization to two standard spaces (MNI152NLin2009cAsym, MNI152NLin6Asym) was performed through nonlinear registration with antsRegistration (ANTs 2.3.3), using brain-extracted versions of both T1w reference and the T1w template. The following templates were selected for spatial

normalization: ICBM 152 Nonlinear Asymmetrical template version 2009c [6, RRID:SCR_008796; TemplateFlow ID: MNI152NLin2009cAsym], FSL's MNI ICBM 152 non-linear 6th Generation Asymmetric Average Brain Stereotaxic Registration Model [7, RRID:SCR_002823; TemplateFlow ID: MNI152NLin6Asym],

Functional data preprocessing

For each of the 3 BOLD runs found per subject (across all tasks and sessions), the following preprocessing was performed. First, a reference volume and its skull-stripped version were generated using a custom methodology of fMRIPrep. Susceptibility distortion correction (SDC) was omitted. The BOLD reference was then co-registered to the T1w reference using bbregister (FreeSurfer) which implements boundary-based registration [8]. Co-registration was configured with six degrees of freedom. Head-motion parameters with respect to the BOLD reference (transformation matrices, and six corresponding rotation and translation parameters) are estimated before any spatiotemporal filtering using mcflirt (FSL 5.0.9) [9]. The BOLD time-series (including slice-timing correction when applied) were resampled onto their original, native space by applying the transforms to correct for head-motion. These resampled BOLD time-series will be referred to as preprocessed BOLD in original space, or just preprocessed BOLD. The BOLD time-series were resampled into standard space, generating a preprocessed BOLD run in MNI152NLin2009cAsym space. First, a reference volume and its skull-stripped version were generated using a custom methodology of fMRIPrep. Automatic removal of motion artifacts using independent component analysis (ICA-AROMA) [10] was performed on the preprocessed BOLD on MNI space time-series after removal of non-steady state volumes and spatial smoothing with an isotropic, Gaussian kernel of 6mm FWHM (full-width half-maximum). Corresponding "non-aggresively" denoised runs were produced after such smoothing. Additionally, the "aggressive" noise-regressors were collected and placed in the

corresponding confounds file. Several confounding time-series were calculated based on the preprocessed BOLD: framewise displacement (FD), DVARS and three region-wise global signals. FD was computed using two formulations following Power (absolute sum of relative motions, [11]) and Jenkinson (relative root mean square displacement between affines, [9]). FD and DVARS are calculated for each functional run, both using their implementations in Nipype (following the definitions by [11]). The three global signals are extracted within the CSF, the WM, and the whole-brain masks. Additionally, a set of physiological regressors were extracted to allow for component-based noise correction (CompCor, [12]). Principal components are estimated after high-pass filtering the preprocessed BOLD time-series (using a discrete cosine filter with 128s cut-off) for the two CompCor variants: temporal (tCompCor) and anatomical (aCompCor). tCompCor components are then calculated from the top 2% variable voxels within the brain mask. For aCompCor, three probabilistic masks (CSF, WM and combined CSF+WM) are generated in anatomical space. The implementation differs from that of Behzadi et al. in that instead of eroding the masks by 2 pixels on BOLD space, the aCompCor masks are subtracted a mask of pixels that likely contain a volume fraction of GM. This mask is obtained by dilating a GM mask extracted from the FreeSurfer's aseg segmentation, and it ensures components are not extracted from voxels containing a minimal fraction of GM. Finally, these masks are resampled into BOLD space and binarized by thresholding at 0.99 (as in the original implementation). Components are also calculated separately within the WM and CSF masks. For each CompCor decomposition, the k components with the largest singular values are retained, such that the retained components' time series are sufficient to explain 50 percent of variance across the nuisance mask (CSF, WM, combined, or temporal). The remaining components are dropped from consideration. The head-motion estimates calculated in the correction step were also placed within the corresponding confounds file. The confound time series derived from head motion estimates and global signals were expanded with the inclusion

of temporal derivatives and quadratic terms for each [13]. Frames that exceeded a threshold of 0.5 mm FD or 1.5 standardised DVARS were annotated as motion outliers. All resamplings can be performed with a single interpolation step by composing all the pertinent transformations (i.e. head-motion transform matrices, susceptibility distortion correction when available, and co-registrations to anatomical and output spaces). Gridded (volumetric) resamplings were performed using antsApplyTransforms (ANTs), configured with Lanczos interpolation to minimize the smoothing effects of other kernels (Lanczos 1964). Non-gridded (surface) resamplings were performed using mri_vol2surf (FreeSurfer).

Supplementary References

1. Tustison, N. J. *et al.* N4ITK: improved N3 bias correction. *IEEE Trans Med Imaging* **29**, 1310–1320 (2010).
2. Avants, B. B., Epstein, C. L., Grossman, M. & Gee, J. C. Symmetric diffeomorphic image registration with cross-correlation: evaluating automated labeling of elderly and neurodegenerative brain. *Med Image Anal* **12**, 26–41 (2008).
3. Zhang, Y., Brady, M. & Smith, S. Segmentation of brain MR images through a hidden Markov random field model and the expectation-maximization algorithm. *IEEE Trans Med Imaging* **20**, 45–57 (2001).
4. Dale, A. M., Fischl, B. & Sereno, M. I. Cortical Surface-Based Analysis: I. Segmentation and Surface Reconstruction. *Neuroimage* **9**, 179–194 (1999).
5. Klein, A. *et al.* Mindboggling morphometry of human brains. (2017) doi:10.1371/journal.pcbi.1005350.
6. Fonov, V. *et al.* Unbiased average age-appropriate atlases for pediatric studies. *Neuroimage* **54**, 313–327 (2011).
7. Evans, A. C., Janke, A. L., Collins, D. L. & Baillet, S. Brain templates and atlases. *Neuroimage* **62**, 911–922 (2012).
8. Greve, D. N. & Fischl, B. Accurate and robust brain image alignment using boundary-based registration. *Neuroimage* **48**, 63–72 (2009).
9. Jenkinson, M., Bannister, P., Brady, M. & Smith, S. Improved optimization for the robust and accurate linear registration and motion correction of brain images. *Neuroimage* **17**, 825–841 (2002).
10. Pruim, R. H. R., Mennes, M., Buitelaar, J. K. & Beckmann, C. F. Evaluation of ICA-AROMA and alternative strategies for motion artifact removal in resting state fMRI. *Neuroimage* **112**, 278–287 (2015).

11. Power, J. D. *et al.* Methods to detect, characterize, and remove motion artifact in resting state fMRI. *Neuroimage* **84**, 320–341 (2014).

12. Behzadi, Y., Restom, K., Liau, J. & Liu, T. T. A component based noise correction method (CompCor) for BOLD and perfusion based fMRI. *Neuroimage* **37**, 90–101 (2007).

13. Satterthwaite, T. D. *et al.* An improved framework for confound regression and filtering for control of motion artifact in the preprocessing of resting-state functional connectivity data. *Neuroimage* **64**, 240–256 (2013).

CCs_Measures

Sub	System	Age	nbCC	Height	Width
Sub-001	M	7.83	19	135.58	10.947
Sub-002	M	11.17	19	106	11.053
Sub-003	M	5.4	16	175.25	11.375
Sub-004	M	7.83	27	52.63	9.6667
Sub-005	T	7.83	15	91.667	9.8667
Sub-006	T	10.33	31	65.323	7.6129
Sub-007	T	7	19	104.95	8.8421
Sub-008	T	12.08	21	109	8.8095
Sub-009	M	11.83	7	146.57	9.8571
Sub-010	M	6.25	13	172	10.154
Sub-011	M	12.08	15	89.2	7.6667
Sub-012	M	10.58	26	111.35	8
Sub-013	M	8.75	14	132.36	15.786
Sub-014	M	5.75	21	57.952	10.095
Sub-015	M	10.17	22	71.636	8.9545
Sub-016	M	7.83	18	97.889	10.5
Sub-017	M	14.58	49	41.49	10.286
Sub-018	M	7.2	21	58	8.8095
Sub-019	M	11.1	22	75.136	10.409
Sub-020	T	9.3	27	56.778	13.37
Sub-021	T	9.3	43	70.581	8.3023
Sub-022	M	7.92	19	123.21	9.5263
Sub-023	M	8.4	37	74.405	9.1892
Sub-024	T	9.75	10	89.1	12.9
Sub-025	M	4.6	26	66.308	11.038
Sub-026	T	8	9	133.56	11.667
Sub-027	T	6.8	48	41.792	8.8542
Sub-028	T	12.17	32	65.844	12.656
Sub-029	T	10	25	49.16	11.64
Sub-030	T	11.6	29	58.069	7.4483
Sub-031	T	7.08	9	62.222	8
Sub-032	T	6	32	64.719	8
Sub-033	T	5.2	27	73.889	10.074
Sub-034	M	13	24	83.25	11.583
Sub-035	T	10.58	43	62.349	8.4651
Sub-036	T	7.58	21	141.48	9.8095
Sub-037	T	9.42	32	82.281	9.9688
Sub-038	T	7.6	10	106.6	12
Sub-039	M	9.3	26	70.731	10.385
Sub-040	M	7.8	30	77.667	8.3333

Sub-041	M	13.4	24	92.375	10.917
Sub-042	T	9.2	22	85.636	9.5
Sub-043	T	11.3	8	122	10.375
Sub-044	T	10.17	25	107.88	9.4
Sub-045	T	11.58	13	111.85	9.3846
Sub-046	M	12.8	21	54.524	13.429
Sub-047	M	8.25	25	77.2	9.64
Sub-048	M	8.8	25	112.84	12.12
Sub-049	M	7.67	17	130.53	11.824
Sub-050	T	8.58	16	166.31	10.875
Sub-051	T	9.5	35	57.029	11.6
Sub-052	M	9.08	40	51	12.625
Sub-053	M	10.83	33	53.606	13.455
Sub-054	T	15.2	25	58.8	10.32
Sub-055	T	12.8	13	74.385	10.615
Sub-056	T	12.8	38	49.5	10.868
Sub-057	T	12.75	16	77.688	13.125
Sub-058	T	8.5	38	45.842	10.316
Sub-059	M	18	32	65.031	9.3438
Sub-060	M	15.4	31	42.194	9.9032
Sub-061	M	12.67	19	70.421	11.263
Sub-062	M	12.3	21	93.143	10
Sub-063	M	10.75	25	41.28	8.76
Sub-064	T	8	11	66.727	10.182
Sub-065	T	7.3	41	54.951	9.0244
Sub-066	M	8.75	38	43.053	11.711
Sub-067	M	7.3	37	47.514	11.973
Sub-068	T	9.42	11	74.818	13.818
Sub-069	M	9.2	37	49.541	10.405
Sub-070	T	11.5	27	56.444	8.4444
Sub-071	M	8.5	13	43.692	8
Sub-072	M	9.7	19	84.053	12.474
Sub-073	T	13.8	23	93.652	12.652
Sub-074	T	7.3	20	49.65	10.9
Sub-075	T	14.8	35	35.686	10.657
Sub-076	M	6.6	9	80.889	16
Sub-077	M	8.5	32	65.125	8.3438
Sub-078	T	7.8	38	51.711	9.8684
Sub-079	T	15.8	25	53.8	11.68
Sub-080	T	17.2	32	42.281	9.2812
Sub-081	T	15.1	37	45.514	9.3514
Sub-082	T	6.7	14	78.643	11.286

Sub-083	T	8.8	30	57.067	11.067
Sub-084	T	11.9	32	72.688	9.25
Sub-085	T	9.3	29	69.828	11.862
Sub-086	M	13.3	24	76.875	10.792
Sub-087	M	15.5	14	55.429	10.286

**Group Montessori SD values**		**Group Traditional SD values**	
Functional System		Functional System	
VIS	2.346280485	VIS	2.209919431
SM	2.382217972	SM	2.357820091
DA	2.437266205	DA	2.401475776
VA	2.400364161	VA	2.37533354
LIM	2.299136419	LIM	2.260144079
FP	2.435327229	FP	2.354722444
DM	2.414775945	DM	2.359996045

**Group Montessori STD values**		**Group Traditional STD values**	
Functional System		Functional System	
VIS	0.647069352	VIS	0.714692553
SM	0.686503116	SM	0.709180182
DA	0.64619527	DA	0.694291416
VA	0.703187945	VA	0.695945521
LIM	0.726823685	LIM	0.737272574
FP	0.666497035	FP	0.709564321
DM	0.656720404	DM	0.696346786
